# Divisive Inhibition Prevails During Simultaneous Optogenetic Activation of All Interneuron Subtypes in Mouse Primary Visual Cortex

**DOI:** 10.3389/fncir.2019.00040

**Published:** 2019-05-28

**Authors:** Tony G. J. Ingram, Jillian L. King, Nathan A. Crowder

**Affiliations:** Department of Psychology and Neuroscience, Dalhousie University, Halifax, NS, Canada

**Keywords:** mouse, vision, interneuron, orientation tuning, primary visual cortex, V1, electrophysiology, optogenetics

## Abstract

The mouse primary visual cortex (V1) has become an important brain area for exploring how neural circuits process information. Optogenetic tools have helped to outline the connectivity of a local V1 circuit comprising excitatory pyramidal neurons and several genetically-defined inhibitory interneuron subtypes that express parvalbumin, somatostatin, or vasoactive intestinal peptide. Optogenetic modulation of individual interneuron subtypes can alter the visual responsiveness of pyramidal neurons with distinct forms of inhibition and disinhibition. However, different interneuron subtypes have potentially opposing actions, and the potency of their effects relative to each other remains unclear. Therefore, in this study we simultaneously optogenetically activated all interneuron subtypes during visual processing to explore whether any single inhibitory effect would predominate. This aggregate interneuron activation consistently inhibited pyramidal neurons in a divisive manner, which was essentially identical to the pattern of inhibition produced by activating parvalbumin-expressing interneurons alone.

## Introduction

**Figure 1:**
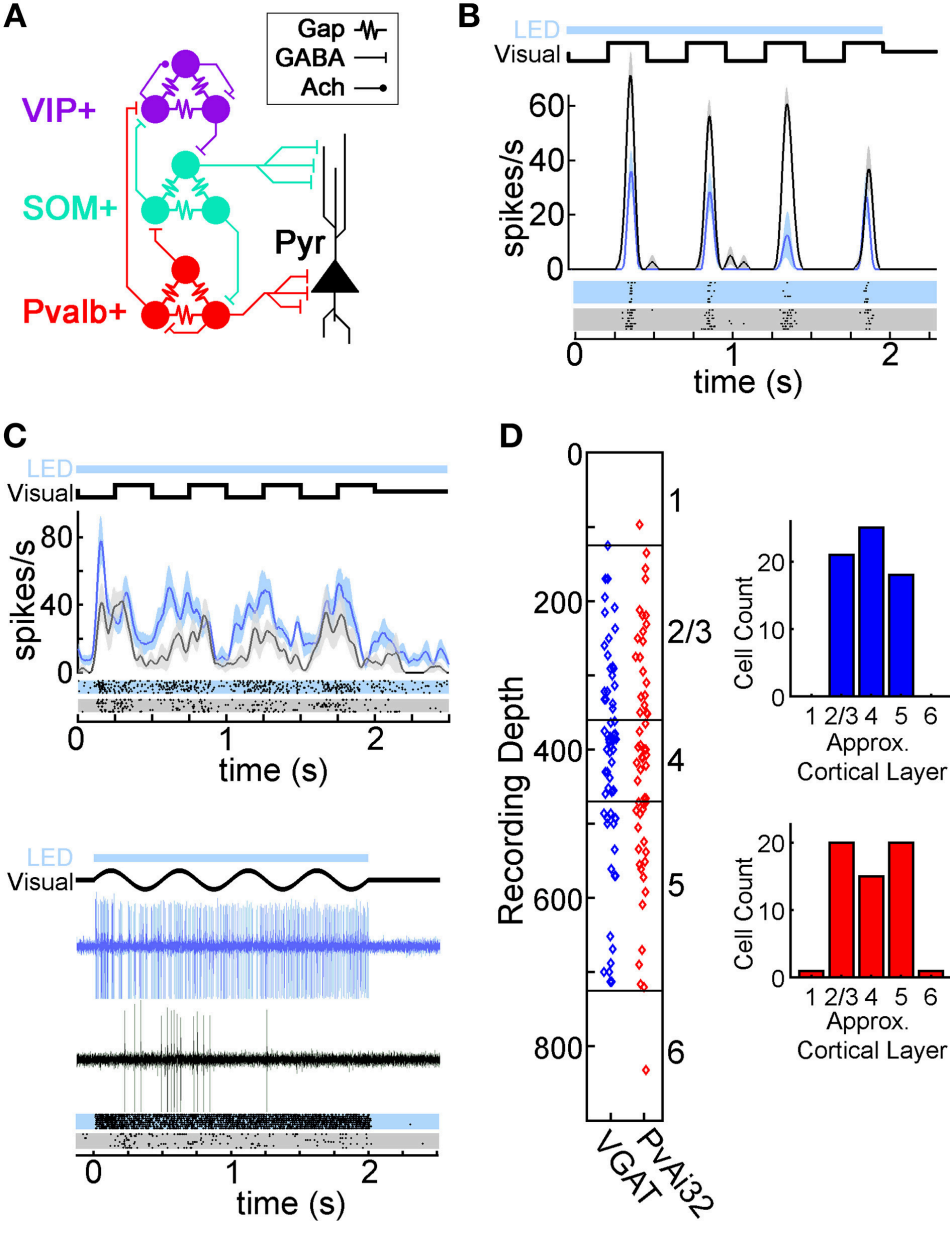
Optogenetic modulation of V1 neurons in transgenic mice. **(A)** Diagram of the proposed wiring of V1 local interneuron circuits described using *in vitro* methods (Pfeffer et al., 2013; Crandall and Connors, 2016; Karnani et al., 2016a). Pyramidal neurons (Pyr; black), and interneurons expressing parvalbumin (Pvalb+; red), somatostatin (SOM+; teal), and vasoactive intestinal peptide (VIP+; purple) are connected with electrical synapses (Gap) as well as GABAergic (GABA) and cholinergic (Ach) chemical synapses. **(B)** Spike Density Functions (SDFs) and rasters for a putative pyramidal neuron's response to drifting square wave gratings with (azure) or without (black) LED illumination. **(C)** Two photostimulated Pvalb+ neurons recorded in PvAi32 mice in the same format as **(B)**. The SDFs and rasters for the top neuron show low intensity photostimulation elevated firing while maintaining important temporal features of visually evoked responses (see Methods), as well as eliciting several low latency spikes. The spike traces and rasters for the bottom neuron illustrate robust and low latency firing evoked by high intensity photostimulation (which was not used in the main dataset). For the example cells in **(B)** and **(C)**, the timing of photostimulation (LED; light blue bar) and the visual stimulus (Visual; thick black line depicting the change in luminance of a point on the monitor over time) are shown above the SDFs or spike traces. Shaded regions on the SDFs in **(B)** and **(C)** indicate SEM. **(D)** Scatter graphs showing recording depths for VGAT (blue) and PvAi32 (red) transgenic mice. Approximate layer boundaries are indicated on the right vertical axis (Lein et al., 2007). Histograms indicating cell count distribution across approximate cortical layers are shown inset.

Understanding how neural circuits produce perception, memory, and action is a fundamental goal of neuroscience (Jorgenson et al., 2015). The mouse primary visual cortex (V1) has become an important brain area for investigating how local interneuron circuits shape cortical information processing thanks in part to the array of genetic tools available in this species (e.g., Hübener, 2003; Callaway, 2005; Luo et al., 2008; Huberman and Niell, 2011), and the foundation of knowledge from classic work in cats and primates (e.g., Hubel and Wiesel, 1962; Movshon et al., 1978a,b; Carandini et al., 1997; Tong, 2003; Espinosa and Stryker, 2012). Mouse V1 comprises ~80% excitatory pyramidal neurons and ~20% GABAergic interneurons (Meinecke and Peters, 1987; DeFelipe, 2002). These interneurons can be further divided into molecularly distinct subtypes that express parvalbumin (Pvalb+; 35–40%), somatostatin (SOM+; 20–30%), and vasoactive intestinal peptide (VIP+; 15–17%), with the remaining ~20% of interneurons being unclassified (Gonchar et al., 2008; Xu et al., 2010; Pfeffer et al., 2013; Pi et al., 2013). Figure 1A shows the proposed wiring for these interneurons derived from *in vitro* studies: Pvalb+ cells inhibit all cell types including each other; SOM+ interneurons inhibit all cell types except themselves; VIP+ interneurons mainly inhibit SOM+ cells but can also inhibit or excite each other weakly; and interneurons of the same type are interconnected with electrical synapses (Pfeffer et al., 2013; Karnani et al., 2016a).

The frequency and strength of connections likely vary between interneuron types, brain regions, and cortical lamina (Crandall and Connors, 2016), so *in vivo* recordings have also been performed to characterize the functional roles of V1 interneurons during sensory processing. Direct Pvalb+ or SOM+ mediated inhibition of pyramidal cells can take the form of arithmetically distinct operations such as division and subtraction. Divisive inhibition produces proportionally greater suppression at higher firing rates, which can scale or normalize the responses of neurons while maintaining their selectivity (Carandini and Heeger, 1994; Salinas and Thier, 2000). For example, the contrast invariant orientation tuning of V1 neurons has been modeled with divisive normalization (Somers et al., 1995; Anderson et al., 2000). Subtractive inhibition produces similar decrements at all firing rates, which due to the spike threshold non-linearity can sharpen selectivity of neurons and narrow sensory tuning (Lee et al., 2012; Wilson et al., 2012). Optogenetic modulation of Pvalb+ interneurons scales pyramidal visual responses divisively whereas SOM+ modulation can shift pyramidal responses subtractively, although these effects appear to depend on both the timing and intensity of optogenetic photostimulation (Atallah et al., 2012, 2014; Lee et al., 2012, 2014; Wilson et al., 2012; El-Boustani and Sur, 2014; El-Boustani et al., 2014). Disinhibition, where one class of interneuron inhibits other types, has also been functionally demonstrated for the projection of VIP+ neurons onto SOM+ cells (Fu et al., 2014; Pakan et al., 2016), and for SOM+ cells inhibiting Pvalb+ cells (Cottam et al., 2013). Within V1's densely interconnected network it is difficult to predict how all these effects might interact (Ayzenshtat et al., 2016). Therefore, our study took a complementary approach by simultaneously optogenetically activating all interneuron types during visual processing to explore whether any pattern of modulation would dominate when the various inhibitory and disinhibitory ensembles compete with each other. We also examined the effects of optogenetically activating Pvalb+ interneurons alone to control for potential differences produced by our anesthetic, visual stimulus, or photostimulation procedures compared to previous work. We found that the orientation tuning of putative V1 pyramidal neurons showed a similar pattern of divisive scaling whether all interneuron types were activated simultaneously or Pvalb+ interneurons were activated alone.

## Materials and Methods

### Animals

All experimental procedures were conducted in accordance with the guidelines of the Canadian Council on Animal Care and were approved by the Dalhousie University Committee on Laboratory Animals. Electrophysiological recordings were collected from 6 VGAT-ChR2(H134R)-EYFP transgenic mice (JAX stock # 014548, Jackson Laboratories), and 13 Pvalb-IRES-Cre;Ai32 mice, which were produced by cross-breeding Pvalb-IRES-Cre (JAX stock # 008069) and Ai32 (JAX stock # 012569) animals. For brevity VGAT-ChR2(H134R)-EYFP and Pvalb-IRES-Cre;Ai32 transgenic mice will henceforth be referred to as VGAT and PvAi32, respectively. Mice were 2–8 months old, and weighed between 20 and 33 g. The neuronal orientation tuning data presented here for the first time was part of a larger set of experiments performed in these mice, parts of which have already been published (King et al., 2016).

### Physiological Preparation

Mice were first pre-medicated with chlorprothixene (5 mg/kg I.P.; Sigma Aldrich), then placed in a custom face-mask and anesthetized with isoflurane in oxygen for the remainder of the experiment (2.5% isoflurane during induction, 1.5% during surgery, and 0.5% during recording; Pharmaceutical Partners of Canada). Additional doses of chlorprothixene were given every 4 h. Anesthetized mice were maintained at a body temperature of 37.5°C with a heating pad. The skull was stabilized with a head-post secured using dental epoxy. V1 was exposed for recording and optogenetic photostimulation with a small craniotomy (~1 mm^2^) 0.8 mm anterior and 2.3 mm lateral from lambda (Paxinos and Franklin, 2001). A wall of petroleum jelly surrounding the craniotomy was filled with saline to prevent dehydration of the cortex. The corneas were protected by frequent application of optically neutral silicone oil (30,000 cSt, Sigma Aldrich). The pupils were not dilated so as to maintain a large depth of focus, and the eyes were not immobilized because eye movements under anesthesia have been shown to be negligible in mice (Wang and Burkhalter, 2007; Niell and Stryker, 2008; Gao et al., 2010).

Extracellular recordings were obtained with glass micropipettes containing 2M NaCl with a tip diameter of 2–5 μm. Electrode depth along vertical penetrations was controlled using a micromanipulator (FHC, Bowdoin, ME). Signals were bandpass filtered between 50 and 2,000 Hz, and sampled at 40 kHz with a CED 1401 digitizer and Spike2 software (Cambridge Electronic Designs, Cambridge, UK). Online analyses were performed in Spike2 from triggered transistor-transistor logic (TTL) pulses from a window discriminator (Cornerstone by Dagan). Spike sorting was performed offline with Spike2 software, which searched for and sorted spikes using a supervised template-matching algorithm. We then used a principle components analysis to check the clustering of spike waveforms.

### Optogenetic Photostimulation

Optogenetic activation of V1 interneurons came about in different ways in the two kinds of transgenic mice. The VGAT transgenic mice express Channelrhodopsin 2 [ChR2(H134R)-EYFP] in all GABAergic neurons (Zhao et al., 2011), and immunohistochemical confirmation of transgene expression in cortex revealed >93% of ChR2(H134R)-EYFP positive neurons were labeled with antibodies against glutamate decarboxylase (GAD67; Zhao et al., 2011), or GABA (King et al., 2016). PvAi32 mice express ChR2(H134R)-EYFP only in Pvalb+ interneurons (Madisen et al., 2012). In these mice, immunohistochemical confirmation of transgene expression in cortex revealed 99% of Pvalb-Cre expressing cells were labeled with antibodies against parvalbumin (Pfeffer et al., 2013), and strong Cre-induced expression of transgene mRNA was found in Pvalb+ neurons (Madisen et al., 2012).

The tip of the fiberoptic cannula was positioned ~0.2–0.5 mm above the surface of V1, and a 470 nm fiber-coupled LED was used for optogenetic photostimulation (0.4 mm diameter; 0.39 NA; Thor Labs). LED activation was coordinated with visual stimuli by the CED 1401 (Figures 1B,C). ChR2(H134R)-EYFP expressing neurons pass measurable photocurrent at a light intensity of ~0.02 mW/mm^2^, which saturates at ~1 mW/mm^2^ (Asrican et al., 2013). Our fiber power output of 0.089–1.16 mW (median: 0.092 mW) was estimated to yield 0.14–1.8 mW/mm^2^ (median: 0.15 mW/mm^2^) at 0.8 mm cortical depth (Stujenske et al., 2015), which is sufficient light intensity to induce photocurrents in ChR2(H134R)-EYFP expressing interneurons even in layer 6. However, since photostimulation intensity was always strongest near the cortical surface it was important to consider the layer distribution of interneurons in V1. No GABAergic interneurons in layer 1 express Pvalb, and few express SOM (~2%) or VIP (~5%). In layers 2/3 a similar proportion of interneurons express VIP (~20%) and Pvalb (~20%), with fewer expressing SOM (8%). In deeper layers ~50% of interneurons express Pvalb, 20–30% express SOM, and ~7% express VIP (Xu et al., 2010; Pfeffer et al., 2013). Thus, our photostimulation was sufficient to activate a large population of Pvalb+ interneurons in PvAi32 mice, and most if not all interneuron subtypes in VGAT mice. We ensured identical photostimulation parameters had no effect on neural firing in wildtype C57BL6J mice that did not express any optogenetic proteins, illumination from the visual stimulus was too dim to inadvertently activate ChR2(H134R)-EYFP expressed in the retina, and that transgenic mice had normal visually guided behavior (King et al., 2016).

Like previous work (Atallah et al., 2012; Lee et al., 2012), we used low to moderate photostimulation intensities to modulate responses over a range where tuning curve shape was retained, which was checked online. Stronger photoactivation of all GABAergic neurons or Pvalb+ cells alone can silence pyramidal cells completely (Atallah et al., 2012; King et al., 2016). Our photostimulation was continuous (rather than eliciting time locked spikes with high intensity flashed photostimulation) to maintain potentially important temporal features of pyramidal and interneuron visual responses such as onset transients, firing rate decay over time, or phase preference for the visual stimulus (Figures 1B,C). Neurons that were activated by photostimulation at low latencies (< 20 ms) irrespective of the visual stimulus were excluded from further analyses because it was likely these cells expressed ChR2(H134R)-EYFP themselves (e.g., Figure 1C; Atallah et al., 2012).

### Visual Stimuli

The receptive fields of isolated visually responsive units were mapped using hand-driven light bars and spots. Quantitative stimuli programmed in MATLAB using the Psychophysics Toolbox extension (Brainard, 1997; Pelli, 1997) were then presented within the classical receptive field on a calibrated CRT monitor (LG Flatron 915FT Plus 19 inch display, 100 Hz refresh, 1024 × 768 pixels, mean luminance = 30 cd/m^2^) at a viewing distance of 15–30 cm. Orientation and direction tuning was measured with full contrast square-wave gratings that drifted in 16 randomly interleaved directions for 2s each trail (fundamental spatial frequency = 0.03 cycles per degree; temporal frequency = 2 Hz). During photostimulated trials LED illumination occurred for the full 2s trial (Figure 1B). Grating stimuli were presented in a circular aperture surrounded by a gray field of mean luminance for 8–12 repetitions. A gray of mean luminance was presented during each 3s inter-trial interval.

### Data Analysis

Spike arrival times were exported to MATLAB (Math Works, Natick, MA) and neuronal responses were represented as spike density functions with 1 kHz resolution, generated by convolving a delta function at each spike arrival time with a Gaussian window. Tuning curves for each neuron were fit to double von Mises functions using the least squares method:

(1) $$\begin{array}{r} \begin{array}{c} R=\;A_{1}e^{\sigma_{1}\left(\cos\left(\frac{\pi }{180}\left(\theta -\phi_{1}\right)\right)-1\right)}+A_{2}e^{\sigma_{2}\left(\cos\left(\frac{\pi }{180}\left(\theta -\phi_{2}\right)\right)-1\right)}+B\; \end{array} \end{array}$$

where *R* is the response in spikes/second; θ is the drift angle of the visual stimulus in degrees; *A*_1_and *A*_2_ are the amplitudes of each peak; σ_1_and σ_2_ are the width constants; ϕ_1_and ϕ_2_ are the centers of each peak; and *B* is the baseline firing rate. Goodness of fit to the curves was measured using *r*^2^. An inclusion criterion of *r*^2^ > 0.5 ensured only tuning curves that were reasonably smooth and orientation tuned were analyzed further. Width constants were converted to half-width at half-height (HWHH; Chang et al., 2012) for more intuitive interpretation:

(2) $$\begin{array}{r} \begin{array}{c} HWHH=\cos^{-1}\left[\frac{\ln\left({\frac{1}{2}e}^{\sigma }+{\frac{1}{2}e}^{-\sigma }\right)}{\sigma }\right] \end{array} \end{array}$$

Orientation selectivity index (OSI) was calculated for each neuron as 1—circular variance (Atallah et al., 2012), where circular variance was calculated as (Ringach et al., 1997):

(3) $$\begin{array}{r} \begin{array}{c} Circular\;Variance=1-\;\frac{\left|\sum_{k}R_{k}e^{i2\frac{\pi }{180}\theta_{k}}\right|}{\sum_{k}R_{k}}\; \end{array} \end{array}$$

where *R*_*k*_ is the neuron's response to orientation *k*. Direction selectivity index (DSI) was calculated for each neuron using the amplitudes from equation 1 (Atallah et al., 2012):

(4) $$\begin{array}{r} \begin{array}{c} DSI=\frac{A_{1}-A_{2}}{A_{1}+A_{2}} \end{array} \end{array}$$

### Modeling GABAergic Inhibition

We modeled the effect of photostimulation on orientation tuning as either divisive or subtractive inhibition. To this end we fit each neuron's response during photostimulation (LEDon) to a double von Mises function where the parameters described for equation 1 were held constant using the estimated parameters from control responses (LEDoff), but with one added term to model either divisive scaling:

(5) $$\begin{array}{r} \begin{array}{c} R_{LEDon}=\frac{R_{LEDoff}}{g} \end{array} \end{array}$$

where g is the scaling term, or subtractive shifting with rectification:

(6) $$\begin{array}{r} \begin{array}{c} R_{LEDon}=R_{LEDoff}-h,\;R_{LEDon}\geq 0 \end{array} \end{array}$$

where h is the shifting term. Each model was fit using the least squares method. Fitted models were compared by calculating an F statistic where the number of parameters are equal (Motulsky and Ransnas, 1987):

(7) $$\begin{array}{r} \begin{array}{c} F=\frac{SS_{\text{DIV}}}{SS_{\text{SUB}}} \end{array} \end{array}$$

where *SS*_*DIV*_ and *SS*_*SUB*_ are the sum of squared residuals between the observed LEDon data and the divisive or subtractive model fits, respectively. For a given neuron a positive F value smaller than 1 indicated a superior fit for the divisive model, and an *F* value > 1 indicated a superior fit for the subtractive model.

### Statistical Analysis

We used parametric and nonparametic analyses where appropriate (specific tests noted in *Results*), and applied the Benjamini-Hochberg procedure for controlling false discovery rate (20 total comparisons). Adjusted *p*-values are reported (Benjamini and Hochberg, 1995).

## Results

We investigated the relative weighting of various GABAergic ensembles in shaping the orientation tuning of V1 neurons by comparing the effects of optogenetically modulating all GABAergic interneuron types simultaneously (in VGAT mice) vs. Pvalb+ cells alone (in PvAi32 mice). All analysis was confined to putative pyramidal neurons, which all showed inhibition to photostimulation. We present data from 64 neurons recorded in VGAT mice, and 57 cells recorded in PvAi32 mice. Electrode depths indicate layers 2–5 were evenly sampled in both VGAT and PvAi32 mice (Figure 1D). Recording depth was poorly correlated with the strength of optogenetic modulation (VGAT: *r* = 0.24; PvAi32: *r* = 0.14) and photostimulation intensity (VGAT: *r* = 0.13; PvAi32: *r* = −0.11), so we made no attempt to segregate optogenetic effects by cortical layer. All subsequent figures organize data from VGAT mice in the left column (blue symbols), and data from PvAi32 mice in the right column (red symbols).

**Figure 2:**
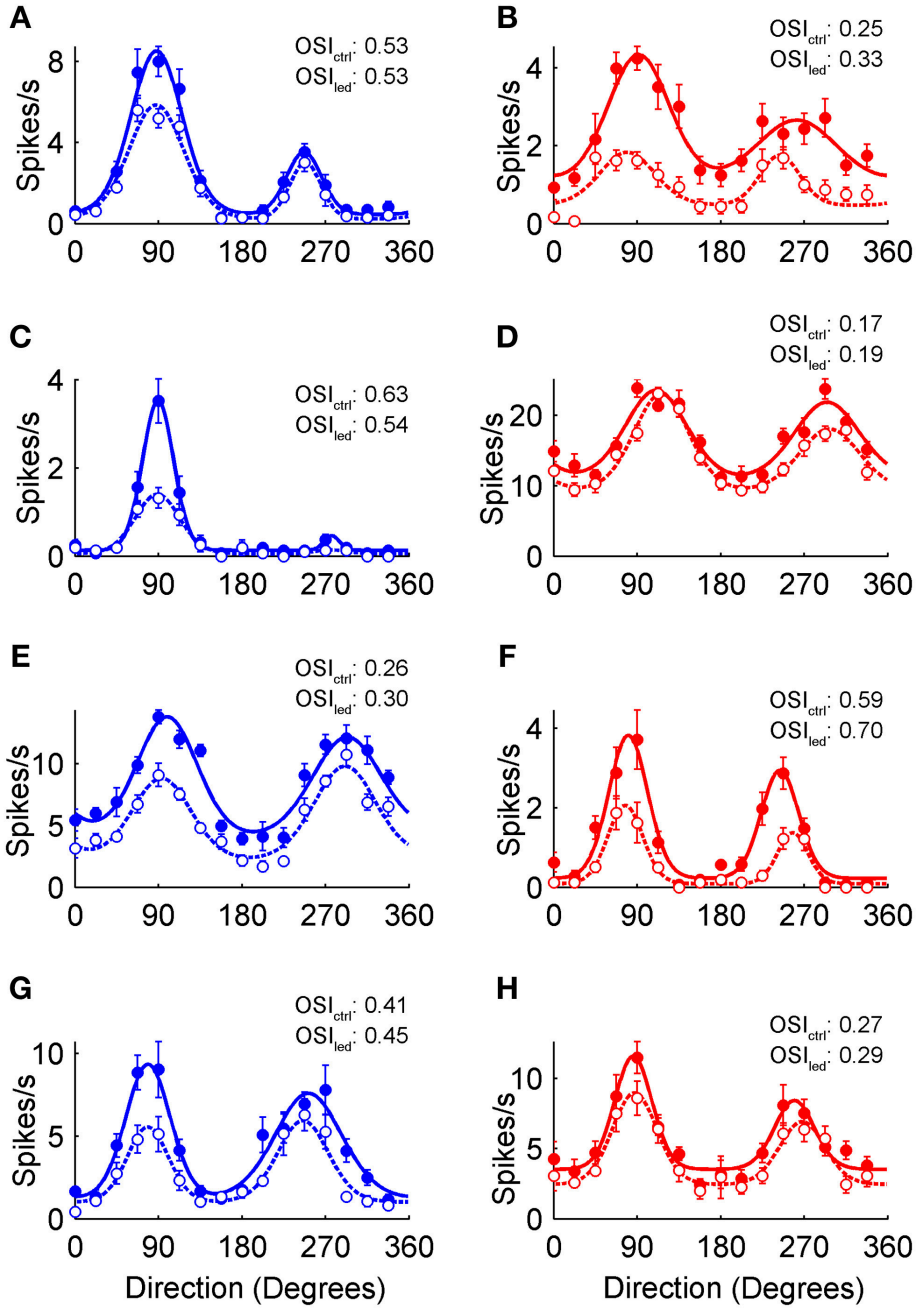
Optogenetic modulation of V1 orientation tuning curves. **(A–H)** Orientation tuning curves from putative V1 pyramidal neurons recorded in VGAT (blue) and PvAi32 (red) transgenic mice. Solid and empty circles show mean firing rates for the control and photostimulated conditions, respectively. Smooth curves show double von Mises fits to control (solid lines) and photostimulation data (dotted lines). Error bars indicate SEM. The orientation selectivity indexes for control (OSI_ctrl_) and photostimulated (OSI_led_) conditions are noted inset.

The sample neurons in Figure 2 show the spectrum of orientation and direction tuning in the control condition (filled circles), as well as the general features of optogenetically induced inhibition (empty circles) observed in VGAT (Figures 2A,C,E,G), and PvAi32 mice (Figures 2B,D,F,H). The preferred direction of each neuron was normalized to 90° for clarity. The smooth curves in Figure 2 show double von Mises fits to the control (solid lines) and photostimulated (dotted lines) data that we used to quantitatively compare the inhibition produced by activating all interneuron types or Pvalb+ cells alone.

### Population Measures of GABAergic Inhibition

**Figure 3:**
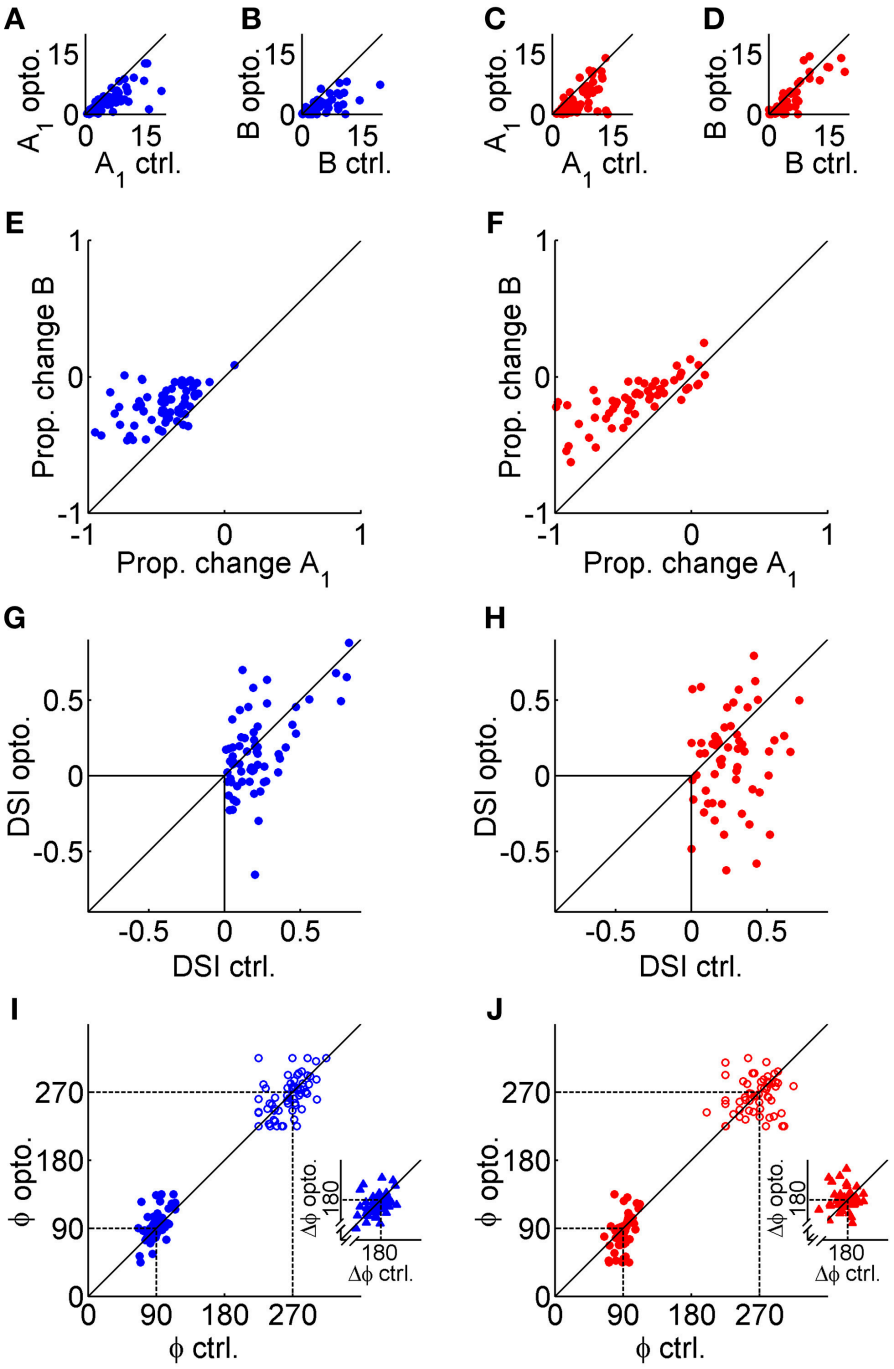
Population data obtained from double von Mises fits. The left column (blue symbols) shows data from VGAT mice, and the right column (red symbols) shows data from PvAi32 mice. **(A,C)** Scatter plots comparing peak firing (A_1_) in the control (ctrl.; abscissa) and photostimulated (opto.; ordinate) conditions. **(B,D)** Scatter plots in a similar format comparing baseline firing (B) in the ctrl. and opto. conditions. **(E,F)** Scatter plots comparing the proportional change in peak (abscissa) and baseline firing (ordinate). Note that photostimulation consistently produced larger drops in peak firing relative to baseline firing. **(G,H)** Scatter plots comparing the direction selectivity index (DSI) calculated in the control (abscissa) and photostimulated (ordinate) conditions. **(I,J)** Scatter plots comparing the preferred direction (ϕ) of the primary (filled circles) and secondary (empty circles) peaks in the control (abscissa) and photostimulated (ordinate) conditions. Inset scatter plots show the peak-to-peak distance (Δϕ) for control (abscissa) and photostimulated (ordinate) conditions.

The effects of GABAergic inhibition on V1 tuning curves were characterized across the population as changes in several parameters extracted from control and photostimulated double von Mises curve fits. Changes in pertinent parameters were analyzed with mixed repeated-measures ANOVAs to examine the main effect of photostimulation and the interaction effect between photostimulation and genotype. None of the measures discussed below showed significant interactions (*p* > 0.05), indicating that there was no evidence that the magnitude of any main effects differed in data obtained from the two mouse genotypes. Both types of mice showed a significant decrease in peak amplitude (*A*_1_) with photostimulation [Figures 3A,C; F_(1, 119)_ = 95, *p* < 6.8 × 10^−16^]. Baseline firing (*B*) also decreased significantly in both types of mice [Figures 3B,D; F_(1, 119)_ = 61, *p* < 1.4 × 10^−11^]. Most of the sample neurons in Figure 2 showed a greater decrease in peak firing than baseline activity, and this difference was significant in the population data from both types of mice [Figures 3E,F; *F*_(1, 119)_ = 195, *p* < 1.6 × 10^−25^]. We found no evidence that photostimulation consistently changed tuning width, as measured with HWHH, for either the primary [*F*_(1, 119)_ = 3.1, *p* = 0.14] or secondary peaks [*F*_(1, 119)_ = 0.14, *p* = 0.94]. Direction selectivity (DSI) decreased significantly with photostimulation [Figures 3G,H; *F*_(1, 119)_ = 19, *p* < 9.6 × 10^−5^]. This effect appeared to arise from the substantial number of negative DSI values in the photostimulation condition, which indicated a reversal in preferred direction. However, these reversals in direction preference appeared to be quite subtle on the tuning curves themselves (e.g., Figures 2B,E,G) because most of these cells were directionally biased (0 < DSI < 0.5) rather than strongly directional (DSI > 0.5). There was no evidence that preferred orientation was changed during photostimulation [*F*_(1, 119)_ = 0.34, *p* = 0.8], and both the main (filled circles) and secondary peak locations (empty circles) clustered tightly along the line of equality in the scatter plots in Figures 3I,J. Orientation tuned or directionally biased V1 neurons are expected to have peaks separated by ~180°, but we did not constrain the peak locations in our double von Mises curves (ϕ_1_, ϕ_2_) to determine whether photostimulation could affect the distance between peaks. As expected, the median peak separation pooled across genotypes was 178° in the control condition. There was no evidence that photostimulation consistently altered the peak-to-peak distance [Figures 3I,J insets; *F*_(1, 119)_ = 0.045, *p* = 0.96].

**Figure 4:**
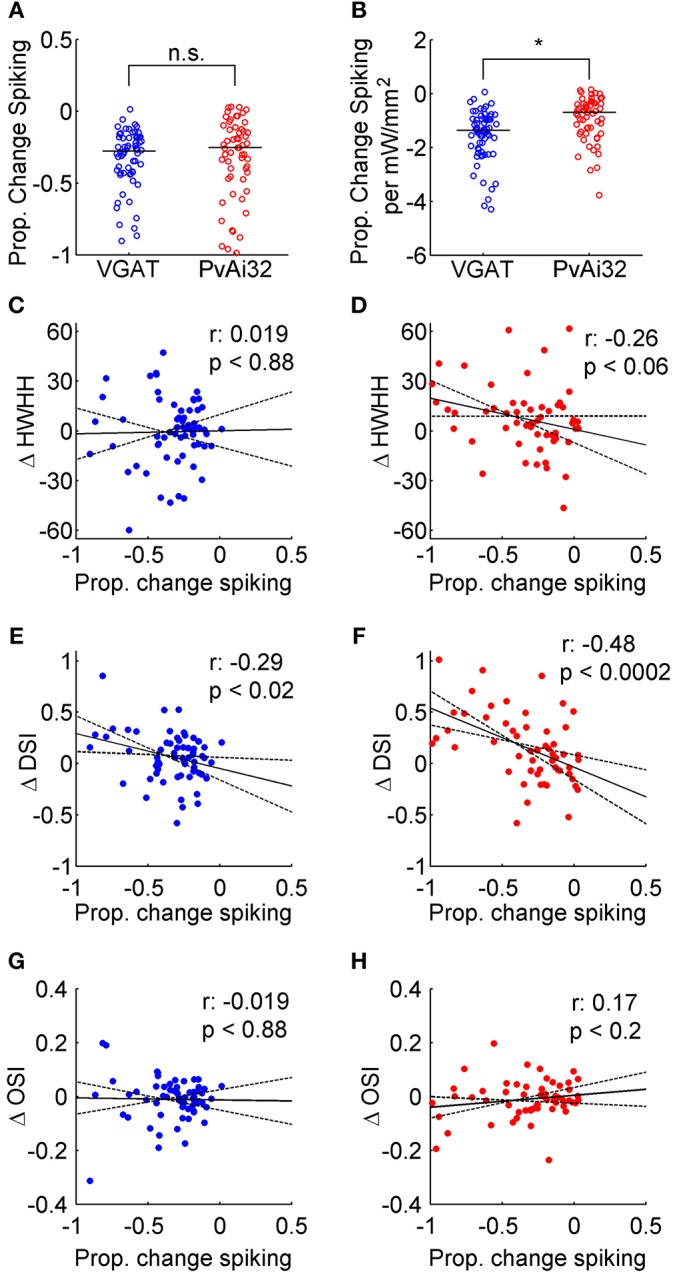
Correlating optogenetically induced suppression with tuning curve changes. Blue symbols show data from VGAT mice and red symbols show data from PvAi32 mice. **(A)** Scatter column graphs comparing the proportional decrease in firing for VGAT and PvAi32 mice. **(B)** Scatter column graphs in a similar format to **(A)**, but the proportional drop in firing was normalized by photostimulation irradiance. The population medians are shown as horizontal lines in **(A)** and **(B)**, and significant (*) and non-significant statistical comparisons (n.s.) are indicated. **(C**,**D)** Scatter plots correlating the proportional decrease in firing induced by photostimulation (abscissa) with the change in tuning breadth (ΔHWHH; ordinate). **(E**,**F)** Scatter plots correlating the proportional decrease in firing (abscissa) with the change in direction selectivity index (ΔDSI; ordinate). **(G**,**H)** Scatter plots correlating the proportional decrease in firing (abscissa) with the change in orientation selectivity index (ΔOSI; ordinate). All correlation scatter plots (**C**–**H**) show the linear regression (solid line), the 95% confidence intervals for the regression (dotted lines), the correlation coefficient (r; top row inset) and *p*-value (bottom row inset).

We quantified the magnitude of suppression as the proportional decrease in firing to the preferred direction induced by photostimulation. We aimed to suppress firing by about a quarter with our photostimulation, which equated to a decreased rate of only 1–3 spikes/s for most neurons. Nonetheless, there was enough variability that a minority of neurons from both VGAT (21%) and PvAi32 (16%) mice had more than −0.5 suppression, which allowed the effects of stronger inhibition to be examined as well. There was no evidence of a difference between the median proportional decrease in firing for VGAT (−0.28) and PvAi32 (−0.25) mice (Figure 4A; Wilcoxon Rank Sum, *p* = 0.68). However, the proportional decrease in firing per mW/mm^2^ of photostimulation irradiance was significantly greater for VGAT mice (Figure 4B; Wilcoxon Rank Sum, *p* < 1.5 × 10^−4^), indicating dimmer photostimulation was able to produce the desired level of suppression when all interneuron types were simultaneously activated.

It has been reported that the magnitude of optogenetically induced inhibition can affect the pattern of changes observed in V1 orientation tuning curves (El-Boustani and Sur, 2014; El-Boustani et al., 2014; Lee et al., 2014), so we correlated tuning curve changes with our measure of suppression. The change in tuning breadth (HWHH) with photostimulation was poorly correlated with the magnitude of suppression in both kinds of mice (Figures 4C,D; *r*-values inset). Conversely, the change in DSI with photostimulation showed a weak to moderate correlation with the magnitude of suppression in both kinds of mice (Figures 4E,F; *r*-values inset). These correlations appear to be driven by two distinct effects. First, many of the aforementioned reversals in direction preference occurred under moderate suppression. Second, there were a few neurons where stronger suppression flattened responses in the anti-preferred direction (*A*_2_) so that DSI increased substantially. The change in orientation selectivity with photostimulation, as measured with OSI, was poorly correlated with the magnitude of suppression in both kinds of mice (Figures 4G,H; *r*-values inset). We included this measure to compare with previous work (e.g., Atallah et al., 2012), however in our hands changes in OSI were noisy because suppression in neurons with little spontaneous firing caused OSI to decrease (Figure 2C) or remain unchanged (Figure 2A), whereas suppression in neurons with even moderate untuned responses in their tuning curves caused OSI to increase (Figures 2B,F). Overall, photostimulation appeared to produce a reliable constellation of changes to orientation tuning, regardless of whether inhibition arose from optogenetically driving just Pvalb+ cells or all interneuron types together.

### Model Fits

Previous work has indicated that different types of interneurons in V1 can either produce a divisive scaling or subtractive shift in pyramidal cell orientation tuning, with the majority of studies suggesting that Pvalb+ neurons induce divisive scaling (Atallah et al., 2012, 2014; Lee et al., 2012, 2014; Wilson et al., 2012; El-Boustani and Sur, 2014; El-Boustani et al., 2014). Several of the photostimulation effects we describe above in PvAi32 mice are consistent with Pvalb+ mediated inhibition taking the form of divisive scaling, but importantly we also observed similar effects in VGAT mice. First, divisive scaling should produce smaller decreases in baseline (*B*) compared to peak firing (*A*_1_), and this is exactly what we observed (Figures 3E,F). Second, divisive scaling is not expected to substantially narrow tuning breadth (HWHH), and this is what we observed as well (Figures 4C,D).

**Figure 5:**
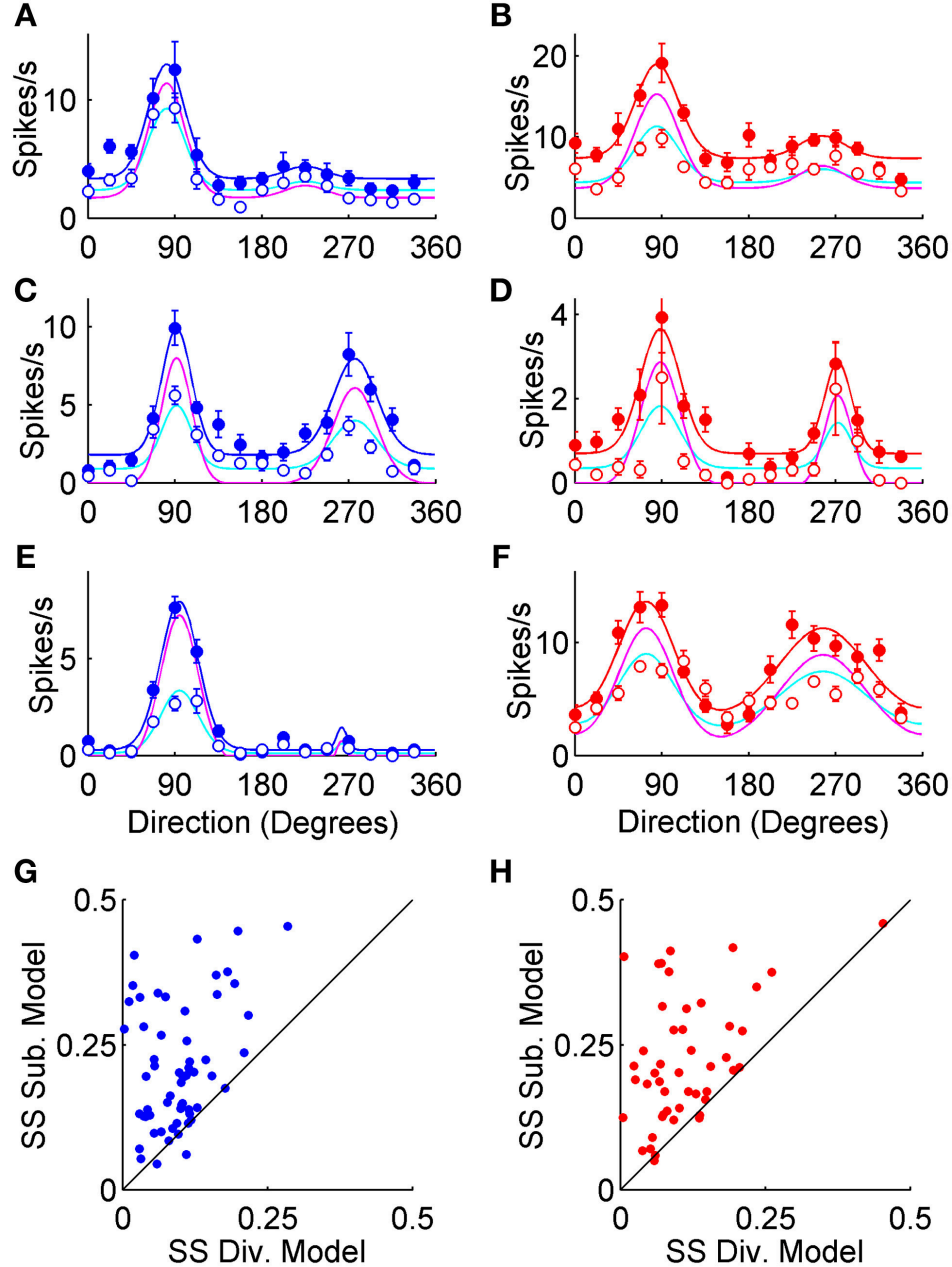
Divisive and subtractive model fits to tuning curves. The left column (blue symbols) shows data from VGAT mice, and the right column (red symbols) shows data from PvAi32 mice. **(A–F)** Orientation tuning curves from putative V1 pyramidal neurons. As in Figure 1, solid and empty circles show mean firing rates for the control and photostimulated conditions, respectively. Control data was fit with double von Mises curves, as shown with smooth blue and red lines in VGAT and PvAi32 transgenic mice, respectively. For each neuron, photostimulated data points were fitted with models that either divisively scaled (cyan curves) or subtractively shifted (magenta curves) the control curve. **(G**,**H)** Scatter plots comparing the sum of squared errors (SS) for the divisive (Div.; abscissa) and subtractive (Sub.; ordinate) model fits to the photostimulated data. Note that the sum of squared errors for the subtractive model were consistently larger than for the divisive model indicating the divisive model provided better fits.

To compare divisive and subtractive inhibition most directly in our data sets, we used each neuron's control double von Mises curve to generate a divisively scaled model and a subtractively shifted model to fit its photostimulated data (see Methods). The subtractive model was rectified to avoid producing negative firing rates. For the example orientation tuning curves shown in Figures 5A–F, the data points as well as the control double von Mises curve fits have an identical format to Figure 2. However, in Figure 5 models of divisive and subtractive inhibition are shown for each cell as cyan and magenta curves, respectively. The divisive model fit the photostimulated data better than the subtractive model for most example neurons because the subtractive model tended to overshoot peak responses and undershoot baseline responses (e.g., Figures 5A–C,F). We normalized all neurons by their maximal control firing rate and then calculated the sum-of-squared residuals for each model. Larger sum-of-squared residuals indicated poorer curve fits, and *SS*_*SUB*_ was larger than *SS*_*DIV*_ for 61/64 (95%) neurons recorded in VGAT (Figure 5G), and 53/57 (93%) neurons recorded in PvAi32 mice (Figure 5H). A mixed repeated-measures ANOVA examining the main effect of photostimulation and the interaction between photostimulation and genotype indicated the divisive model provided significantly better fits to the photostimulated data than the subtractive model [*F*_(1, 119)_ = 92, *p* < 1.1 × 10^−15^], and there was no evidence of an interaction [*F*_(1, 119)_ = 0.03, *p* = 0.96]. Furthermore, the subtractive model did worse with greater levels of suppression: the F-statistic comparing the two model fits (see equation 7 in *Methods*) was strongly correlated with the optogenetically induced proportional decrease in firing to the preferred direction in both VGAT (*r* = 0.82; *p* < 1 × 10^−5^) and PvAi32 mice (*r* = 0.66; *p* < 1 × 10^−5^). Overall, the model fitting supported previously published reports that Pvalb+ interneurons provide divisive inhibition to pyramidal neurons (Atallah et al., 2012; Wilson et al., 2012; El-Boustani and Sur, 2014; El-Boustani et al., 2014), but also indicated that this divisive inhibition prevails when all interneurons are activated simultaneously.

## Discussion

Mouse V1 has a densely interconnected network structure where different subclasses of interneurons not only inhibit pyramidal cells, but also inhibit each other to produce disinhibition of pyramidal neurons (Figure 1A; Pfeffer et al., 2013; Karnani et al., 2016a). Here we measured the orientation tuning of putative pyramidal neurons while all interneuron types were simultaneously optogenetically activated as one way of exploring the functional balance among the wide variety of potentially opposing GABAergic connections. Pyramidal neurons consistently showed divisive scaling during this aggregate activation, which was essentially identical to the effect of photostimulating Pvalb+ neurons alone (Atallah et al., 2012, 2014; Wilson et al., 2012; El-Boustani and Sur, 2014; El-Boustani et al., 2014; Lee et al., 2014).

Our results help establish the relative weighting of several modulatory effects described with *in vivo* studies that targeted subclasses of interneurons individually. Both Pvalb+ and SOM+ interneurons provide direct inhibition to pyramidal cells (Figure 1A), however multiple lines of evidence indicate they can have different effects. First, Pvalb+ cells mainly target the perisomatic regions of pyramidal neurons, whereas SOM+ cells target pyramidal cell dendrites (Freund and Buzsaki, 1996; Markram et al., 2004; Tremblay et al., 2016). These distinct innervation patterns suggest different roles for Pvalb+ and SOM+ interneurons in V1 because it has been proposed (mainly from hippocampal data) that interneurons that innervate pyramidal cell dendrites modulate the plasticity of specific inputs that terminate in the same dendritic domain, whereas interneurons that target the perisomatic region control pyramidal cell output and can synchronize action potentials in cell populations (Cobb et al., 1995; Miles et al., 1996; Freund and Katona, 2007). Second, recent work showed inhibition in V1 pyramidal cells could be either divisive or subtractive depending largely on the intensity and timing of Pvalb+ or SOM+ photostimulation relative to their target cells (Atallah et al., 2012, 2014; Lee et al., 2012, 2014; Wilson et al., 2012; El-Boustani and Sur, 2014; El-Boustani et al., 2014). Our mild to moderate photostimulation intensity caused Pvalb+ interneurons to scale pyramidal activity divisively in our PvAi32 sample (Figure 5H; Atallah et al., 2012; Wilson et al., 2012; Lee et al., 2014), so it is likely that Pvalb+ neurons acted similarly when we activated all interneuron types in our VGAT mice. SOM+ interneurons can provide subtractive inhibition when their activation lags that of pyramidal cells (Wilson et al., 2012; El-Boustani and Sur, 2014); however our relatively large grating stimuli and photostimulation over the full trial likely caused SOM+ interneurons to switch to divisive inhibition as predicted by El-Boustani and Sur (2014). Therefore, pyramidal neurons in the VGAT mice were probably scaled divisively by direct inhibition from both Pvalb+ and SOM+ interneurons.

Photostimulation consistently produced inhibition in our VGAT mice suggesting inhibition to pyramidal cells generally outweighed disinhibition. We found the lack of disinhibition surprising for several reasons. First, VIP+ interneurons are most abundant in layers 2/3 and should have been robustly photostimulated in our VGAT mice. The disinhibitory projection from VIP+ to SOM+ interneurons has been extensively explored (Lee et al., 2013; Pfeffer et al., 2013; Pi et al., 2013; Karnani et al., 2016a,b), and evidence suggests it plays a role in the modulatory effect of locomotion (Fu et al., 2014) and attention (Zhang et al., 2014). Perhaps when all interneurons were simultaneously activated this VIP+ disinhibition was counteracted by inhibitory projections onto VIP+ neurons from the more numerous Pvalb+ and SOM+ interneurons (Figure 1A). Second, SOM+ interneurons have been shown to inhibit Pvalb+ interneurons at least twice as potently as they inhibit pyramidal cells (Cottam et al., 2013), but this may have merely biased the source of direct inhibition to pyramidal cells in favor of SOM+ neurons rather than producing disinhibition. Importantly, this bias would be undetectable if both SOM+ and Pvalb+ inhibition was divisive as proposed above.

Although our approach proved useful for probing *in vivo* interactions among interneuron ensembles, several limitations of this work highlight pathways forward for future investigations. The first limitation is that during natural viewing and behavior it is unlikely that all interneuron types would be activated with identical timing or intensity for a prolonged block of time. Future work could investigate the importance of timing of interneuron activity within this circuit if various cell types could be targeted separately by having them express optogenetic proteins actuated by different wavelengths of light (Prigge et al., 2012; Wietek and Prigge, 2016). Our homogenous photostimulation across the cortical surface is another limitation because neural activity, even within a single interneuron type, varies across V1 with the retinotopic representation of stimulus features and possibly with cortical layer as well. Future work could utilize laser photostimulation at the scale of single neurons to explore spatial interactions among various interneuron types (Fu et al., 2014; Karnani et al., 2016b), although this method is itself limited to relatively superficial cortical layers due to scattering of light by neural tissue (Yizhar et al., 2011; Stujenske et al., 2015; Yona et al., 2016). In summary, divisive inhibition dominates V1 during aggregate photostimulation of GABAergic interneurons, but efforts to further disentangle the interactions among distinct interneuron ensembles will likely require more nuanced control of optogenetic actuators and photostimulation.

## Ethics Statement

All experimental procedures were conducted in accordance with the guidelines of the Canadian Council on Animal Care and were approved by the Dalhousie University Committee on Laboratory Animals.

## Author Contributions

NC: designed the study. JK and NC: collected data. TI, NC, and JK: analyzed the data and wrote the manuscript.

### Conflict of Interest Statement

The authors declare that the research was conducted in the absence of any commercial or financial relationships that could be construed as a potential conflict of interest.
